# MicroRNAs as New Players for Diagnosis, Prognosis, and Therapeutic Targets in Breast Cancer

**DOI:** 10.1155/2009/305420

**Published:** 2009-07-27

**Authors:** Enders K. O. Ng, Chris L. P. Wong, Edmond S. K. Ma, Ava Kwong

**Affiliations:** ^1^Department of Surgery, The University of Hong Kong, Hong Kong; ^2^Department of Molecular Pathology, Hong Kong Sanatorium & Hospital, Hong Kong

## Abstract

MicroRNAs are small nonprotein-coding RNAs that regulate the expressions of a wide variety of genes by sequence-specific base pairing on the 3′UTR of mRNA targets resulting in mRNA degradation or inhibition of translation. Aberrant expressions of miRNAs have been linked to tumor development, metastasis, diagnosis, prognosis, and therapy response in human breast cancer. Some miRNAs have been considered to have potential clinical applications as a tool for breast cancer prognosis and therapy. Here we describe and discuss lines of evidence supporting the important relationship between miRNAs and breast cancer, and its therapeutic strategies.

## 1. Introduction

Recently, the discovery of a class of small nonprotein-coding RNAs, so-called microRNAs (miRNAs), has opened new opportunities in cancer biology. MiRNAs are 19–25-nucleotides regulatory nonprotein-coding RNA molecules that regulate the expressions of a wide variety of genes by sequence-specific base pairing on the 3′ untranslated regions (3′UTR) of the target mRNA resulting in mRNA degradation or inhibition of translation. Patterns of miRNA expression are meticulously regulated and play important roles in oncogenesis [1, 2]. In the last decade, the number of human genes known to be regulated by miRNAs is growing rapidly [3, 4]. Increasing numbers of studies showed that miRNA expression correlates with various cancers and thought to function as both tumor suppressors and oncogenes. Downregulation or accumulation of subsets of miRNAs implies a tumor suppressor or oncogenic function, respectively, is often seen in tumor development, as in the examples of downregulated let-7 in lung cancer [5], deleted or downregulated miR-15 and miR-16 in chronic lymphocytic leukemia [6], and miR-17-5p and miR-20a control the balance of cell death and proliferation [7]. To date more than 700 human miRNAs are annotated in the miRBase registry (miRBase version 12.0), but most of the genes regulated by human miRNAs are not well defined. These miRNAs are also predicted to regulate 30% protein-coding genes in the human genome, indicating their importance as global regulators in gene expression [8]. In this review, we will focus on recent findings of miRNAs related to breast cancer development and explore the potential usefulness of miRNAs for the diagnosis, prognosis, and potential therapeutic targets of breast cancer.

## 2. Aberrant Expression of miRNAs in Human Breast Cancer

Over recent years, miRNA expression studies, especially large-scale profiling, have been providing certain detailed overview that aberrant expression of miRNAs is associated with human breast cancer. By using high-density microarray approaches, a set of significant deregulated miRNAs has been revealed in breast tumors compared to normal breast tissues [9–11]. Lu et al. [11] reported that miRNA expression globally downregulated in tumors, including breast cancer, compared to normal tissues, and demonstrated that miRNA signature can classify human cancers according to developmental lineage and differentiation status more accurate than mRNA expression profiles. The first report describing breast cancer-specific miRNA profiling on a large set of tumor and normal breast tissues identified a list of 29 differentially expressed miRNAs and was able to discriminate tumors from normal tissues with high accuracy [9]. Among the differentially expressed miRNAs in breast cancer, miR-10b, miR-125b, miR-145, miR-21, and miR-155 were revealed to be the most consistently deregulated. The downregulation of miR-10b, miR-125b, and miR-145 and upregulation of miR-21 and miR-155 suggested that these miRNAs could play a role as tumor suppressor genes or oncogenes. A recent study used miRNA profiling to classify the subtypes of breast tumors and identified a number of miRNAs associated with molecular subtypes of breast cancer and some miRNAs correlated with clinicopathological parameters [12]. Intriguingly, another recent report connected four miRNAs (miR-7, miR-128a, miR-210, and miR-51-3p) to breast cancer progression of Estrogen receptor (ER) positive and lymph node negative [13].

## 3. Functional Effects of miRNAs and Targets

The involvement of miRNAs in cancer etiology is emerging because of their capacity to directly target gene transcripts and influence cellular physiology. Since miRNAs can explicate their function via regulation of specific mRNAs, there has been a great interest in identifying their targets.

Among the miRNAs identified as potential oncogene, miR-21 is one of the best evaluated miRNAs. Overexpression of miR-21 has been identified commonly in solid tumors of the lung, breast, stomach, prostate, colon, brain, head and neck, esophagus and pancreas [10, 14–16]. In breast cancer, suppression of miR-21 both in vitro and in vivo led to increase in apoptosis and downregulated the antiapoptotic factor Bcl-2 [15]. Another potential gene targeted by miR-21 in breast cancer was Tropomyosin 1 (TPM1), which play an important role in antioncogenic function including binding microfilaments and regulating cytoskeleton miRNAs [17]. Several potential target genes of miR-21 have been identified including the tumor suppressor PTEN, and the protein programmed cell death 4 (PDCD4) [18, 19]. These findings suggest that miR-21 acts as an oncogene in breast cancer development.

miR-10b is another miRNA that seems to play a vital role in breast cancer. The expression of miR-10b was found to be associated with clinical progression and metastasis in breast carcinoma [20]. They reported that miR-10b is indeed down modulated in all the breast carcinomas from metastasis-free patients. Nevertheless, half of the patients with metastasis showed elevated miR-10b levels in their primary tumors. This study also revealed that miR-10b regulated homeobox D10, leading to upregulation of the prometastatic gene RHOC and thus result in cell invasion and metastasis.

miR-27a is reported to target the transcription factor ZBTB10/RINZF, which is a putative suppressor of specificity protein (Sp) [21]. Overexpression of Sp by miR-27a contributes to the increased expression of Sp-dependent survival and angiogenic genes, including survivin, vascular endothelial growth factor (VEGF), and VEGF receptor 1 (VEGFR1). Thus, the oncogenic activity of miR-27a in breast cancer cells is due, in part, to suppression of ZBTB10.

Another crucial pathway in breast cancer is estrogen mediated signaling. In one study, it is reported that miR-206 expression is significantly downregulated in estrogen receptor alpha (ER*α*)-positive breast cancer tissues [22]. The potential binding sites of miR-206 was identified in the 3′UTR of human ER*α* gene [22, 23], suggesting a mutually inhibitory feedback loop between ER*α* and miR-206.

miR-17-5p (also known as miR-91), located on chromosome 13q31, seems to exert the role as a tumor suppressor in breast cancer. Study demonstrated that miR-17-5p regulated AIB1 (Amplified in Breast Cancer-1 protein) [24]. AIB1 is a coactivator for nuclear receptors, such as ER. It is overexpressed in breast cancer and can promote breast cancer cell proliferation through an increase in the transactivation of ER. Study also found that the loss of heterozygosity of miR-17-5p genomic location occurred in breast cancer. These findings highlight miR-17-5p act as a tumor suppressor affecting critical molecular events involved in breast cancer progression.

Another miRNA as a potential tumor suppressor in breast cancer is miR-125b or miR-125b. miR-125a and -b were downregulated in breast cancer [9]. Study demonstrated that either miR-125a or miR-125b can suppress the expression of HER2 and HER3 [25]; two important tyrosine kinase receptors frequently deregulated in breast cancer. Restoration of miR-125a/b impaired tumor cell growth and reduced tumor cell migration and invasion, suggesting a therapeutic use of miRNAs in tumor suppression.

Cancers may arise from rare self-renewing tumor-initiating cells (T-IC). However, how T-IC self-renewal, multipotent differentiation, and tumorigenicity are maintained remains unresolved. By comparing miRNA profiling in self-renewing and differentiated cells from breast cancer lines and in breast T-IC (BT-IC) and non-BT-IC from 1 degree breast cancers, let-7 were found to be markedly reduced in BT-IC and increased with differentiation [26]. Restoration of let-7 in BT-IC reduced proliferation, mammosphere formation, and the proportion of undifferentiated cells in vitro and tumor formation and metastasis in NOD/SCID mice, while antagonizing let-7 by antisense oligonucleotides enhanced in vitro self renewal of non-T-IC. Increased let-7 reduced both H-RAS and HMGA2, indicating that both genes are let-7 targets. Therefore let-7 regulates multiple BT-IC stem cell-like properties by silencing more than one target.

Breast cancer metastasis suppressor 1 (BRMS1) is a predominantly nuclear protein that differentially regulates expression of multiple genes, leading to suppression of metastasis without blocking orthotopic tumor growth in multiple human and murine cancer cells of diverse origins. Hurst et al. found that BRMS1 suppresses metastasis via alteration of miR-146 expression and associated with decreased signaling through the nuclear factor-kappaB pathway [27]. Furthermore, they showed that BRMS1 significantly upregulates miR-146a and miR-146b in metastatic breast cancer cells. Transduction of miR-146a or miR-146b downregulated expression of epidermal growth factor receptor, inhibited invasion, and migration in vitro, and suppressed experimental lung metastasis. These findings further support the recent notion that modulating the levels of miR-146a or miR-146b could have a therapeutic potential to suppress breast cancer metastasis. A summary of miRNAs and their target genes associated with breast cancer are listed in Table 1.

## 4. miRNAs as Tools for Diagnosis and Prognosis

Since numerous investigations demonstrated that miRNAs are involved in cancer development and metastasis, miRNAs are now being explored to have great cancer diagnostic and prognostic potentials. Emerging evidence revealed that pattern of miRNAs expression correlated well with clinicopathological characteristics and disease outcome. In a previous study of chronic lymphocytic leukemia, a unique signature of 13 miRNAs was associated with prognostic factors and disease progression [28]. Another study reported that expression signatures of several miRNAs could accurately discriminate acute myeloid leukemia with common translocation [29]. Furthermore, high expression of miR-155 and low expression of let-7a-2 were associated with poor prognosis in human lung cancer [30].

In human breast cancer, several studies revealed that miRNAs act as biomarkers in diagnosis and prognosis [31, 32]. One recent study on 219 early breast cancer patients identified that expression level of miR-210 was inversely correlated with disease-free and overall survival, suggesting that miR-210 could be an independent prognostic factor for breast cancer [33]. Another study using miRNA microarray profiling investigated miRNA expression pattern associated with clinicopathological characteristics and patient survival and revealed that overexpressed miR-21 was associated with specific breast cancer pathological features such as advanced tumor stage, lymph node metastasis, and poor survival [16]. Intriguingly, one recent report identified that miR-7, miR-128a, miR-210, and miR-516-3p were associated with tumor aggressiveness in ER positive and lymph node negative and miR-210 linked with early relapse in ER positive and lymph node negative breast cancer [13]. Furthermore, low levels of miR-335 and miR-126 expression were identified to be strongly correlated with metastatic relapse in breast cancer patients [34].

Although miRNAs have been identified as biomarkers either in cancer cell lines or in biopsy specimens, the invasive nature of a biopsy makes it unsuitable for cancer screening in high-risk populations. It would be desirable to have a method that could accurately detect cancer without resorting to an invasive procedure. Recently, several reports suggest that cell-free circulating miRNAs are detectable in serum/plasma and the levels of tumor-derived miRNAs elevated in the patients with tongue cancer [35], lung cancer [36], prostate cancer [37], ovarian cancer [38], and colorectal cancer [39]. These findings suggest that blood-based miRNAs could emerge as revolutionary sources of biomarker for breast cancer diagnosis.

## 5. Potential Usefulness of miRNA in Breast Cancer Therapy

As emerging evidences highlight the importance of miRNAs in diagnosis and prognosis of breast cancer, the usefulness of miRNA-based breast cancer therapy is now being explored.

MiRNA-based cancer therapy seems to offer an alternative for targeting multiple gene networks that are controlled by a single miRNA. In breast cancer, it would be reasonable to consider miRNAs to be involved in specific networks, such as the HER family-mediated or ER-driven signaling. These pathways are likely to be able to alter the response to chemotherapy or to targeted therapy, such as Trastuzumab (directed against HER2) or Tamoxifen (antioestrogen). The previous findings that miR-206 regulated ER*α* expression in breast cancer [22] support the suggestion that miR-206 could be a novel candidate for endocrine therapy to specifically target ER*α*. Since miR-125a/b was another miRNA involved in HER family-mediated pathway [25], such miRNA could be another target to investigate the mechanism of action of Trastuzumab to the perturbation of signaling pathway in cancer cells.

Another study showed that doxorubicin (DOX)-resistant breast cancer cells exhibited alterations in miRNA profile and miR-451 was identified to regulate the expression of multidrug resistance 1 gene [40]. Further investigation showed that restoring miR-451 into DOX-resistant breast cancer cells resulted in the increased sensitivity of cells to DOX, indicating that restoration of such altered miRNA expression may have important implications for breast cancer therapy.

Interestingly, a recent study demonstrated that estradiol (E_2_) downregulated miR-21 expression and induced miR-21 target genes, such as PDCD4 and PTEN, expression in breast cancer cells [18]. Considering that miR-21 was overexpressed in breast cancer and regulated the tumor suppressor PTEN in breast cancer [18, 19], there would be great interest to target miR-21 so as to regulate PTEN in breast cancer because PTEN has been demonstrated as a modulator of responsiveness to Trastuzumab [41]. Another miRNA which may involve in the Trastuzumab-mediated effects is p27 which is an important cell cycle regulator [42]. As miR-221 and miR-222 have been showed to regulate p27 in different types of cancer [43, 44], the investigation on the relationship between such miRNAs and p27 in breast cancer may provide potential therapeutic use for improvement of responsiveness to Trastuzumab.

HER2 overexpression is a hallmark of a subset of breast cancer aggressiveness, and its activation is strictly dependent on the transinteraction with other members of HER family, in particular, the activation of the PI3K/Akt survival pathway. Interestingly, a recent study showed that miR-205, downregulated in breast tumors compared with normal breast tissue, directly targets HER3 receptor and inhibits the activation of the downstream mediator Akt [26]. HER3 plays an important role in HER2-mediated tumorigenesis, and coexpression of both HER2 and HER3 is a poor prognostic factor. In this study Iorio et al. found that restoration of miR-205 in breast cancer cells increased the responsiveness to tyrosine kinase inhibitors Gefitinib and Lapatinib, abrogating the HER3-mediated resistance and restoring a potent proapoptotic activity. It suggested miR-205 as a new oncosuppressor in breast cancer and can improve the responsiveness to specific anticancer therapies.

Although there is no established model between p53 and miRNAs in breast cancer development, recent intriguing evidence linking p53 and miR-34 family showed that miR-34a is a direct target of p53 [45, 46]. It would be interesting to investigate if miR-34a is deregulated in breast cancer patients carrying wild-type p53. If so, this would be one of the mechanisms used by tumor cells to escape the apoptotic control by p53 and to survive under oncogenic circumstance. Thus, miRNA-based therapy by restoring miR-34a function in breast cancer could also be a strategy to improve responsiveness to chemotherapy.

Apart from chemotherapy, miRNAs have been suggested to contribute to responsiveness to radiotherapy. In a recent study, it showed that the let-7 family of miRNAs can suppress the resistance to radiotherapy in lung cancer cells [47]. These findings are the first direct evidence that miRNAs can suppress resistance to anticancer cytotoxic therapy, a common feature of cancer cells, and suggest that miRNAs may be a viable tool to augment current cancer therapies.

## 6. Concluding Remarks

The past decades have seen advances in the diagnosis and treatment of breast cancer. However, breast cancer is still a leading cause of cancer-related deaths among women, with as many as 40% relapsing with metastatic disease [48]. Despite the dedication of research and resources to the elucidation of molecular mechanisms involved, unpredictable response and development of resistance to adjuvant therapy remain major challenges in breast cancer management. The emergence of microRNAs (miRNAs) as upstream regulators of gene expression is identified as novel candidate diagnostic, prognostic indicators, and therapeutic targets. Due to the advancement of high-throughput miRNA expression profiling, the important involvement of miRNAs in breast cancer development and metastasis has been uncovered. Besides, miRNAs as biomarkers used for breast cancer diagnosis and prognosis in clinical setting have important advantages in contrast to mRNAs: (i) unlike screening for large number of mRNA expression, a modest number of miRNAs may be sufficient to differentiate cancers from normal; and (ii) unlike mRNAs, miRNAs in plasma remain largely intact and have been proven to be more stable for detection in paraffin-embedded tissues [49] and even presented in a remarkably stable form in plasma and serum samples that is protected from endogenous RNase activity [37]. A recent study also shows that an added exogenous miRNA (miR-141) has a slower rate of reduction and remained detectable for longer periods than placental mRNA, suggesting that miRNAs have higher stability than mRNAs even in the absence of any protection (e.g., through association with particles) from nuclease activity in the plasma [50]. Thus, with increasing knowledge about miRNAs associated with molecular subtypes and clinicopathological characteristics of breast cancer we believe that miRNAs may prove useful as diagnostic and prognostic tools in the future.

As the therapeutic implications of miRNAs are emerging, miRNA-based therapeutic strategies can be formulated by either antagonizing or restoring the functions of miRNAs. Antisense oligonucleotide technology is already being developed by using the intrinsic RNA interference (RNAi) pathway for gene therapy. Specific designed miRNA inhibitors or synthetic miRNAs targeting genes involved in breast cancer, such as HER2 [25] has shown some promise of miRNA-based cancer therapy being used as a powerful tool for breast cancer therapy. Although it is promising, two major obstacles must be overcome before it can become a broadly applicable standard therapy: the question of their specificity and efficient delivery to the target cells. RNAi applications are based on base pairing between an oligonucleotide and an RNA. In practice, a single mismatch can lead to a complete loss of silencing [51] and so “off-target effects” compromise the specificity of RNAi if sequence identity between small interfering RNA (siRNA) and random mRNA transcripts causes RNAi to knock down expression of nontargeted genes. If multiple siRNAs are used for targeting multiple hits, multiple off-target effects will happen. Thus, RNAi application will never be completely specific. More recent investigations have shown that by suitable design of the siRNAs as well as the use of modified nucleotides, however, the unspecific effects can be minimized [52–55]. The delivery of miRNA inhibitors or synthetic miRNAs into cells is one of the greatest challenges to the development of this application. Despite the advances of the past few years, further developments are still required to get systemically applied miRNA inhibitors to their required site of action. Although viral vector systems offer additional options for efficient and organ-specific delivery, this approach must first overcome the reservations based on the negative experience with gene therapy.

The specific inhibition of a single target gene is usually attempted, and experience in the antisense field has shown that this can, under some circumstances, be insufficient for complex diseases such as cancer. In contrast, miRNAs affect many target RNAs, so that more comprehensive regulation can be achieved with the inhibition of a single miRNA. Thus, further understanding of the functional roles of miRNAs will open up new opportunities in developing revolutionary therapeutic strategies for breast cancer chemoprevention.

## Figures and Tables

**Table 1:** List of miRNAs and their target gene involved in human breast cancer.

MicroRNA	Targets	Aberrant expression and roles	References
MiR-21	BCL2, TPM1, PDCD4, PTEN	Overexpressed and play oncogenic role	[14–17]
MiR-10b	Homeobox D10	Down modulated, but associated with metastatic potential	[20]
MiR-27a	ZBTB10	Oncogenic role in breast cancer cells	[21]
MiR-206	ER*α*	Overexpressed	[22, 23]
MiR-17-5p	AIB1	Oncosuppressor in breast cell lines	[24]
MiR-125a, miR-125b	ERBB2, ERBB3	Down modulated and act as oncosuppressor	[9, 25]
Let-7	RAS, HMGA2	Down modulated and reduced in BT-ICs	[26]
MiR-205	HER3	Down modulated	[27]
